# Incorporating prior knowledge to facilitate discoveries in a genome-wide association study on age-related macular degeneration

**DOI:** 10.1186/1756-0500-3-26

**Published:** 2010-01-28

**Authors:** Wan-Yu Lin, Wen-Chung Lee

**Affiliations:** 1 Graduate Institute of Epidemiology, College of Public Health, National Taiwan University, Taipei, Taiwan; 2 Research Center for Genes, Environment and Human Health, National Taiwan University, Taipei, Taiwan

## Abstract

**Background:**

Substantial genotyping data produced by current high-throughput technologies have brought opportunities and difficulties. With the number of single-nucleotide polymorphisms (SNPs) going into millions comes the harsh challenge of multiple-testing adjustment. However, even with the false discovery rate (FDR) control approach, a genome-wide association study (GWAS) may still fall short of discovering any true positive gene, particularly when it has a relatively small sample size.

**Findings:**

To counteract such a harsh multiple-testing penalty, in this report, we incorporate findings from previous linkage and association studies to re-analyze a GWAS on age-related macular degeneration. While previous Bonferroni correction and the traditional FDR approach detected only one significant SNP (rs380390), here we have been able to detect seven significant SNPs with an easy-to-implement prioritized subset analysis (PSA) with the overall FDR controlled at 0.05. These include SNPs within three genes: *CFH*, *CFHR4*, and *SGCD*.

**Conclusions:**

Based on the success of this example, we advocate using the simple method of PSA to facilitate discoveries in future GWASs.

## Background

Substantial genotyping data produced by current high-throughput technologies have brought opportunities and difficulties. High-density genotyping platforms have been developed in a hope that underlying disease-associated genes can be identified through denser and denser collections of single-nucleotide polymorphism (SNP) data. However with the number of SNPs going into millions comes the harsh challenge of multiple-testing adjustment. To counteract multiple-testing penalty incurred by testing such a large number of SNPs, some genome-wide association studies (GWASs) responded by taking a large sample size--with the number of study subjects soaring into thousands, tens of thousands, or even more [1].

There are two approaches for multiple-testing adjustments. One is controlling the family-wise error rate (FWER), the other is controlling the false discovery rate (FDR) [2,3]. The FWER is defined as the probability of at least one type I error. Among methods for controlling FWER, the Bonferroni correction is the best known approach, although it is very conservative. Holm's step-down procedure [4] is less conservative than the classical Bonferroni correction. The FWER can also be controlled by the resampling-based *P*-value adjustment procedure. Compared with controlling the FWER, controlling the FDR is usually a more powerful approach. However, even with the FDR approach, a GWAS may still fall short of discovering any true positive gene, particularly when it has a relatively small sample size. When testing simultaneously for a huge number of SNPs, even true positive SNPs would have difficulty in standing out among all the noise, based on a straight (and brutal) comparison of their *p *values. GWAS on age-related macular degeneration (AMD) is a good example, and we will show this in this paper.

The above simple FDR approach has been further extended to dependent tests and to tests with prior information [5]. The false discovery control with *P*-value weighting [5,6] can improve power when the assignment of weights (based on previous linkage evidence [6]) is adequate, but there is some power loss when the weights are poorly assigned. Sun et al.'s [7] stratified false discovery control is another approach. They partitioned all SNPs into two subsets based on minor-allele frequencies (MAFs), and then the FDR control is applied to the two subsets respectively. However, as pointed out by Li et al. [8], MAFs have little relevance with biological information and thus partitioning SNPs based on MAFs probably may not improve much power. To address this issue, Li et al. [8] proposed a 'prioritized subset analysis' (PSA). The PSA makes clever use of available prior knowledge, either of the linkage information, the biological information or both. We will show that the PSA can greatly facilitate discoveries in GWASs, with a demonstration on an AMD data.

## Methods

### Materials: a GWAS on Age-related Macular Degeneration (AMD)

AMD is a genetically complex disorder. The heritability was estimated to range from 46% to 71%. Klein et al. [9] reported an AMD data set containing 96 AMD cases and 50 controls. Of all the 116,204 genotyped SNPs, 99,317 SNPs were informative (MAF ≥ 1%) and conformed to Hardy-Weinberg equilibrium (with Hardy-Weinberg exact *p *value ≥ 0.05 in the 50 controls). Following Klein et al. [9], we test for allelic association with disease status on each SNP.

### Prioritized Subset Analysis

To facilitate discoveries in GWASs, we turned to a new method of 'prioritized subset analysis' (PSA) [8]. To perform a PSA, a researcher based on his/her prior biological knowledge first picks from among all SNPs under study, a certain number of SNPs likely to be the true positives. He/she then places those selected SNPs in a 'prioritized subset' and those remaining in a 'non-prioritized' subset. The FDR control is then applied to these two subsets separately, and the significant results are harvested from both the two subsets.

### Prioritizing SNPs

We took findings from previous genome-wide linkage and association studies on AMD as our prior knowledge to prioritize SNPs. Our prioritization process is detailed below.

We first incorporated evidence of linkage (with LOD score >2.0) based on previous linkage studies [10-16]. We obtained the physical position of each D-number marker (listed in Table 1) from the Gene Location website http://genecards.weizmann.ac.il/geneloc/index.shtml. Then SNPs within 500 kb from each D-number marker were prioritized.

Moreover, several genes have had at least one positive association finding [17,18]. These genes with SNPs genotyped in the AMD data set are listed in Table 1. For example, complement factor H (*CFH*, GeneID 3075) gene within 1q32 was reported to be related to AMD, both from genome-wide linkage analyses [10-15] (all published before Klein et al. [9]) and case-control studies [19-21] (all independent of Klein et al.'s study [9], and published at the same year of Klein et al.'s paper [9]). We also learnt that *LOC387715*/*HTRA1 *(GeneID 5654) locus within 10q26 was identified as a second major locus contributing to AMD pathogenesis [22,23]. Furthermore, complement component factor B (*CFB*, GeneID 629) and the adjacent complement component 2 (*C2*, GeneID 717) on chromosome 6p21, were reported to act along the same biological pathway as *CFH *[24,25]. When prioritizing SNPs, we used the Bioinformatics tool 'GenoWatch' [26] to identify SNPs around these candidate genes. The chromosomal region around the *CFH *gene had been shown by several independent studies to be linked [10-15] or associated [19-21] with AMD, so we prioritized SNPs within 1 Mb from the *CFH *gene. For other candidate genes in Table 1, we prioritized SNPs within 50 kb from each. (For example, to check whether SNP rs800292 is within 1 Mb from the *CFH *gene, we simply insert 'rs800292' into 'SNP ID' and '1 Mb' into the 'Upstream' and 'Downstream' on the website of GenoWatch [26]: http://genepipe.ngc.sinica.edu.tw/genominer/menu.do)

**Table 1:** Genes or markers to be prioritized, in the prioritized subset analysis

Chr.	Genes	D-number markers	No. of SNPs in the prioritized subset
1	*ABCA4, CFH*	*D1S549*	94
2		*D2S1356, D2S1394, D2S1384*	103
3	*CX3CR1*	*D3S1768, D3S1304, D3S3045*	93
4		*D4S2368*	19
5		*D5S820, GATA12A08, D5S1506*	169
6	*HLA, C2-CFB, VEGF, ELOVL4, SOD2*		20
7	*PON1*		10
9	*VLDLR, TLR4*	*D9S930, D9S934*	49
10	*LOC387715/HTRA1*		12
12	*LRP6*		9
19	*APOE*	*D19S245*	32
20	*CST3, MMP9*		17
22		*D22S683*	12

In the end, a total of 639 SNPs were prioritized, and the remaining 98,678 SNPs, non-prioritized. We then applied the PSA with the FDR being controlled at 0.05, for both the prioritized subset and the non-prioritized subset. We used Storey and Tibshirani's [3] smoothing spline approach provided by the package 'fdrtool' [27] to estimate the proportions of true negative SNPs.

## Results

### Bonferroni Correction and Traditional FDR Approach

Controlling the FWER at 0.05 (the level of significance for each SNP being set at 0.05/99,317 = 5.03 × 10^-7 ^with the Bonferroni correction), only one significant SNP (rs380390) can be identified (within the *CFH *gene, see Table 2). (Klein et al. [9] actually found one additional significant SNP, rs10272438, but it was later dropped because of low call rate and possible genotyping errors.) Controlling the FDR at 0.05 wasn't any better--the same (and the only one) SNP rs380390 was found to be significant (Table 2). Note that this SNP, rs380390, though being significant, was still not 100% guaranteed to be a true positive (because it was detected under a FDR control value of 0.05).

**Table 2:** Results of the AMD data set

Chr.	Location (bp)	SNP	*P *value *	Bonferroni	FDR	PSA	Gene
1	193930492	rs800292	5.12 × 10^-4^	NS **	NS	S	*CFH*
1	193962973	rs2019727	3.01 × 10^-4^	NS	NS	S	
1	193989310	rs380390	5.40 × 10^-8^	S	S	S	
1	193991069	rs1329428	3.09× 10^-6^	NS	NS	S	

1	194173603	rs1853882	1.59 × 10^-4^	NS	NS	S	*CFHR4*

5	155782975	rs970476	7.20 × 10^-5^	NS	NS	S	*SGCD*
5	155791718	rs931798	3.69 × 10^-4^	NS	NS	S	

* *P *values were obtained from Fisher's exact test for allelic association with disease status.

** S: significant; NS: not significant

### Prioritized Subset Analysis

The PSA identified a total of seven significant SNPs (all from the prioritized subset) (Table 2). These include SNPs within three genes: *CFH*, *CFHR4*, and *SGCD*. By using the PSA method, we have been able to detect six additional significant SNPs (in two additional genes), compared to the Bonferroni approach (the method used by Klein et al. [9]) or the traditional FDR approach. Two of the three significant genes found in this study, *CFH *and *CFHR4*, are located in a chromosomal region (1q31-1q32) having been most replicated in previous AMD studies. The remaining one significant *SGCD *gene had not been previously reported to be AMD-related, though. However, we notice that previous animal studies showed the *SGCD *gene is related to vascular abnormalities in mice [28]. This might suggest a link of *SGCD *to neovascular AMD in humans.

All the seven significant SNPs are from the prioritized subset. To evaluate how well the FDR is controlled in our prioritized subset, we further estimated the permutation-based FDR [29] in this subset. We randomly permuted the data and calculated the null *P *values -  for the *i*th SNP in the *b*th permutation (*i *= 1,...,639). Through *B *permutations, the number of false positives (*FP*) is estimated as , where *d *= 5.12 × 10^-4 ^is the largest *P *value of the seven significant SNPs (see Table 2). We took *B *= 100,000 and obtained  = 0.225. The permutation-based FDR in the prioritized subset is thus estimated as 0.225/7 = 0.032, which is still less than our FDR control level of 0.05, suggesting a satisfactory FDR control in this subset.

## Discussion

Prior information can come from a researcher's biological knowledge, or findings of data other than that provided in the current study. But one should not 'snoop' his/her data at hand for the prior knowledge. If one naively prioritizes those SNPs with the smallest *p *values in the study data, the actual overall FDR would no longer be properly controlled. To avoid such bias, we searched findings of other data to build our 'prior knowledge', *before *seeing the analysis results of individual SNPs in the current AMD data set. At that time, we did know that rs380390 is a significant SNP in the AMD data set which can withstand a FWER control of 0.05 [9]. But the chromosomal region around rs380390 had already been replicated by many *previous *linkage studies [10-15] (all published before Klein et al. [9]). And so, prioritizing chromosomal region around rs380390 won't constitute an act of data snooping.

Around a particular gene, how large a chromosomal region should be prioritized is also an issue. Because of the consistent findings in the *CFH *gene, both from genome-wide linkage analyses [10-15] and case-control studies [19-21], we prioritized SNPs within 1 Mb from the *CFH *gene. Other evidence of linkage and associations are relatively unconfirmed by prior studies, so we prioritized SNPs within 500 kb and 50 kb, respectively. Because linkage is a coarse mapping whereas association is a fine mapping, in general a wider region of SNPs should be prioritized for a linkage peak. Admittedly, there is no absolute criterion for choosing the sizes of prioritized regions. No matter how large a chromosomal region is prioritized, the FDR within subsets should be controlled at the desired level, and this can be verified by estimating the permutation-based FDR [29].

In recent GWASs, a commonly used approach to incorporate prior knowledge is to calculate the Bayes factors [1,30]. However, to estimate the Bayes factors, the prior distributions and the effect sizes should be carefully specified [30]. This may limit its applicability. By contrast, the PSA method used in this paper can feed on prior knowledge that is only rudimentary (we need only to decide beforehand whether a particular SNP is more likely a true positive or a true negative, but don't need to know exactly how likely). And there is almost no penalty for poor guessing [8]. In this paper, we demonstrated that such a simple dichotomization followed by a simple PSA can greatly facilitate discoveries in a GWAS on AMD.

Note that we did not recruit any more subjects or type any more SNPs beyond what Klein et al. [9] had done. The only thing we did is to incorporate prior knowledge about AMD into the analysis. And we see this input of knowledge is rather powerful (six/two additional significant SNPs/genes were identified in the same AMD case-control data). One may question that our input of knowledge and the subsequent partition of SNPs into two subsets to be tested separately and harvested combinedly are making easier (and perhaps too easier) for the SNPs to come out. But we should emphasize that we did not loosen our FDR control in any way. The total seven significant SNPs found in this re-analysis have an overall 0.05 FDR attached to them, much the same way with the one SNP rs380390 originally found in Klein et al. [9] had a 0.05 FDR attached to it. And we believe that researchers will find no difficulties to choose seven SNPs or just one--that is, under the same FDR criteria.

## Conclusions

The PSA approach is rather powerful and is easy to implement. Based on the success of our re-analysis of Klein et al's GWAS on AMD, we advocate using PSA to facilitate discoveries in future GWASs.

## Competing interests

The authors declare that they have no competing interests.

## Authors' contributions

W-Y L participated in the design of the study, performed the data analyses, and drafted the manuscript. W-C L conceptualized the study, provided advice, and revised the manuscript. All authors read and approved the final manuscript.
